# *Pseudoexeirarthra*, a new genus from New Zealand (Coleoptera, Staphylinidae, Pselaphinae), with descriptions of seven new species

**DOI:** 10.3897/zookeys.491.9164

**Published:** 2015-03-26

**Authors:** Jong-Seok Park, Christopher E. Carlton

**Affiliations:** 1 Louisiana State Arthropod Museum, Department of Entomology, LSB 404, Louisiana State University Agricultural Center, Baton Rouge, LA 70803, U.S.A.

**Keywords:** Taxonomy, biogeography, Faronitae, Faronini, redescription

## Abstract

A new endemic genus and seven new species of New Zealand pselaphine staphylinid beetles of the supertribe Faronitae are described as follows: *Pseudoexeirarthra* Park & Carlton, **gen. n.** (type species: *Sagola spinifer* Broun); *Pseudoexeirarthra sungmini* Park & Carlton, **sp. n.**; *Pseudoexeirarthra kwangguki* Park & Carlton, **sp. n.**; *Pseudoexeirarthra youngboki* Park & Carlton, **sp. n.**; *Pseudoexeirarthra seiwoongi* Park & Carlton, **sp. n.**; *Pseudoexeirarthra parkeri* Park & Carlton, **sp. n.**; *Pseudoexeirarthra hlavaci* Park & Carlton, **sp. n.**; *Pseudoexeirarthra nomurai* Park & Carlton, **sp. n.** Three species, *Sagola spinifer* Broun, *Sagola colorata* Broun, and *Sagola puncticollis* Broun, are transferred to the genus *Pseudoexeirarthra*. Six species are synonymized: *Sagola dilucida* Broun, *Sagola guinnessi* Broun, *Sagola longicollis* Broun, *Sagola longula* Broun, and *Sagola rectipennis* Broun under *Pseudoexeirarthra spinifer* (Broun); *Sagola insueta* Broun under *Sagola colorata* (Broun). A lectotype is designated for *Pseudoexeirarthra spinifer* (Broun). A key, habitus photographs, line drawings of diagnostic characters, and distribution maps are provided for each species.

## Introduction

*Sagola* Sharp, 1874, the largest genus of the supertribe Faronitae, has been considered to be a paraphyletic assemblage of species (Chandler 2001). *Sagola* was recently revised by Park and Carlton (2014) as well as other extant genera, *Exeirarthra* (Park and Carlton 2011) and *Stenosagola* (Park and Carlton 2013). Three species, *Sagola spinifer* Broun, *Sagola puncticollis* Broun, and *Sagola colorata* Broun are distinctive morphologically and can be easily separated from the other *Sagola* species by the absence of anterior and posterior frontal foveae, absence of promesocoxal foveae, presence of an inverted triangle-shaped process along the anterior margins of abdominal tergites IV–VI, and female sternite VIII bearing a pseudosternite. Based on these characters, a new genus, *Pseudoexeirarthra* gen. n. is established to accommodate the three previously described species and seven new species.

## Materials and methods

Approximately four hundred specimens were studied from the Field Museum of Natural History (FMNH), Chicago, IL, USA; Louisiana State Arthropod Museum (LSAM), Baton Rouge, LA, USA; Natural History Museum (NHM), London, United Kingdom; Lincoln University (LUNZ), Lincoln, New Zealand; New Zealand Arthropod Collection (NZAC), Auckland, New Zealand; Auckland Museum, Auckland, New Zealand (AMNZ); personal collection of Donald S. Chandler (DSC), Durham, NH, USA; personal collection of John T. Nunn (JTN), Dunedin, New Zealand.

Holotypes of species described herein are deposited in the New Zealand Arthropod Collection (NZAC), Auckland. Paratype and additional specimen depositions are indicated parenthetically. Specimen label data for types are transcribed verbatim. Data for other specimens are standardized for consistency.

Seven specimens were mounted on permanent slides to aid in observation of internal characters and fine external characters not apparent when using a dissecting microscope. Permanent microscopic slides were prepared using the techniques described by Hanley and Ashe (2003). Terminology for the foveal system and enumeration of abdominal sclerites follows Chandler (2001). Numbering of abdominal sclerites indicates actual segment counts (i.e., not ventrites) for consistency with Chandler’s system, but meso- metathoracic ventral sclerites are referred to as ventrites (*sensu* Beutel and Leschen 2010).

New Zealand maps were produced by modifying the map of Crosby et al. (1976) and adding appropriate symbols using Adobe Photoshop®. The area codes for the New Zealand biotic regions follow the system of Crosby et al. (1998). Multiple specimens from the same locality are indicated by a single symbol.

Each figure of an aedeagus illustrates the organ in dorsal view with the median lobe oriented forward (up on page). Right and left are indicated based on this orientation, not the morphological orientation when inside the body, which would be reversed.

## Taxonomy

### Pseudoexeirarthra

Taxon classificationAnimaliaColeopteraStaphylinidae

Park & Carlton gen. n.

http://zoobank.org/950B4AD1-52D2-4BBD-90A6-4F57AE86FCA5

#### Type species.

*Sagola spinifer* Broun, 1895: 75; here designated.

#### Diagnosis.

Members of *Pseudoexeirarthra* can be separated from those of all other faronite genera by the following combination of characters: body length 1.8–2.8 mm; frontal sulcus broad and shallow, reaching level of the midline of eyes (Fig. 2A); lacking anterior and posterior frontal foveae (Fig. 2A); prosternum with lateral procoxal foveae (Fig. 2B); mesoventrite lacking promesocoxal foveae (Fig. 2C); tergites IV–VI with inverted triangle-shaped process on anterior margins (Fig. 2D); sternites IV–VI with basolateral foveae; female sternite VIII with pseudosternite (Fig. 2F); female sternite IX bearing pair of small process that each bear two long setae (Fig. 2G).

#### Description.

**Figure 1.**
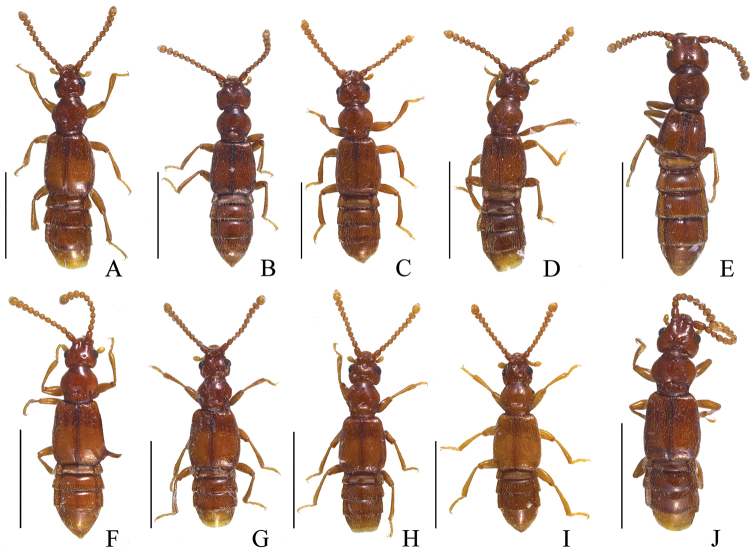
Habiti, dorsal view: **A** *Pseudoexeirarthra spinifer* (Broun) **B** *Pseudoexeirarthra colorata* (Broun) **C** *Pseudoexeirarthra puncticollis* (Broun) **D** *Pseudoexeirarthra sungmini* sp. n. **E** *Pseudoexeirarthra kwangguki* sp. n. **F** *Pseudoexeirarthra youngboki* sp. n. **G** *Pseudoexeirarthra seiwoongi* sp. n. **H** *Pseudoexeirarthra parkeri* sp. n. **I** *Pseudoexeirarthra hlavaci* sp. n. **J** *Pseudoexeirarthra nomurai* sp. n. Scale bars = 1 mm.

**Figure 2.**
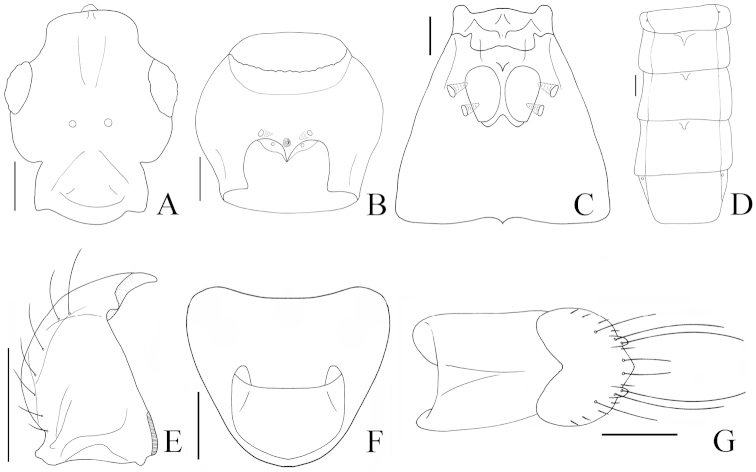
*Pseudoexeirarthra spinifer* (Broun): **A** head, dorsal view **B** prosternum, ventral view **C** meso- and metaventrite, ventral view **D** abdomen, dorsal view **E** left mandible, dorsal view **F** female sternite VIII, dorsal view **G** female sternite IX, ventral view. Scale bars = 0.1 mm.

**Figure 3.**
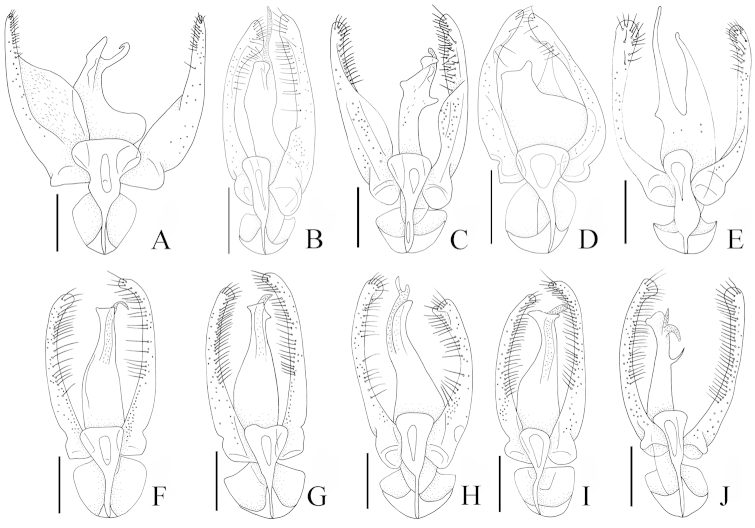
Aedeagi, dorsal view: **A** *Pseudoexeirarthra spinifer* (Broun) **B** *Pseudoexeirarthra colorata* (Broun) **C** *Pseudoexeirarthra puncticollis* (Broun) **D** *Pseudoexeirarthra sungmini* sp. n. **E** *Pseudoexeirarthra kwangguki* sp. n. **F** *Pseudoexeirarthra youngboki* sp. n. **G** *Pseudoexeirarthra seiwoongi* sp. n. **H** *Pseudoexeirarthra parkeri* sp. n. **I** *Pseudoexeirarthra hlavaci* sp. n. **J** *Pseudoexeirarthra nomurai* sp. n. Scale bars = 0.1 mm.

Body length 1.8–2.8 mm. Body reddish, antennae, legs, maxillary palpi and elytra paler (Fig. 1A–J). Head. Antennae gradually clavate, reaching posterior margin of prothorax. Head bluntly triangular and longer than wide (Fig. 1A–J). Apex of left mandible thicker than right (Fig. 2E). Frontal sulcus broad and shallow, reaching level of the midline of eyes, lacking anterior and posterior frontal foveae (Fig. 2A). Prosternum bearing median and lateral procoxal foveae (Fig. 2B). Mesoventrite lacking promesocoxal foveae, bearing lateral mesoventral and lateral mesocoxal foveae (Fig. 2C). Metaventrite with pair of lateral metaventral foveae (Fig. 2C). Tergites IV–VI with inverted triangle-shaped process on anterior margins (Fig. 2D). Aedeagus with bulky and round phallobase (Fig. 3A–J). Parameres bearing setae along mesal margin for one-third to two-thirds length of parameres (Fig. 3A–J). Female sternite VIII with pseudosternite (Fig. 2F). Female sternite IX bearing pair of small process that each bear two long setae (Fig. 2G).

#### Etymology.

The generic name refers to the superficial similarity to the genus *Exeirarthra* Broun.

#### Comments.

Members of this genus lack distinct external secondary sexual characters except on abdominal sternite IX. Male sternite IX is fragile, and partially concealed by sternite VIII, rendering it simple and reduced in appearance. Females possess a more robust, heart-shaped or rounded sternite IX that bears two pairs of long setae that are usually visible in ventral view. Female genitalia, including spermathecae, apparently are membranous and were not observed after clearing specimens using 10% potassium hydroxide.

#### Key to species of *Pseudoexeirarthra* gen. n.

**Note.** The key is largely based on male genitalia because most specimens are indistinguishable based on the external morphology. Antennal shape and eye size are apparently unique within species. However, apparent variations may result from viewing at inconsistent orientations among specimens. These characters are difficult to interpret consistently when performing identifications, but still useful in comparing types or specimens in series.

**Table:** 

1	Elytra as long as wide (Fig. 1E), abdominal tergite IV lacking patch of microtrichia	***Pseudoexeirarthra kwangguki* sp. n.**
–	Elytra longer than wide, abdominal tergite IV with transverse patches of microtrichia	**2**
2 (1)	Left paramere at least twice as wide as right paramere at midpoint (Fig. 3A)	***Pseudoexeirarthra spinifer* (Broun)**
–	Let paramere close to as wide as right paramere at midpoint	**3**
3 (2)	Median lobe of genitalia at least 3 times as wide as either paramere (Fig. 3D)	***Pseudoexeirarthra sungmini* sp. n.**
–	Median lobe of genitalia at most slightly more than twice as wide as either paramere	4
4 (3)	Parameres broader than median lobe of genitalia (Fig. 3C)	***Pseudoexeirarthra puncticollis* (Broun)**
–	Parameres narrower than median lobe of genitalia	**5**
5 (4)	Median lobe of genitalia with acute spine at one-third length (Fig. 3J)	***Pseudoexeirarthra nomurai* sp. n.**
–	Median lobe of genitalia lacking branch	**6**
6 (5)	Apical lobe of genitalia triangular (Fig. 3G)	***Pseudoexeirarthra seiwoongi* sp. n.**
–	Apical lobe of genitalia bluntly rounded	**7**
7 (6)	Left paramere longer than right (Fig. 3H)	***Pseudoexeirarthra parkeri* sp. n.**
–	Right paramere longer than left	**8**
8 (7)	Major apical lobe of genitalia rectangular (Fig. 3B)	***Pseudoexeirarthra colorata* (Broun)**
–	Major apical lobe of genitalia inverted triangular apically	**9**
9 (8)	Major apical lobe of genitalia with slightly wider apical margin (Fig. 3F)	***Pseudoexeirarthra youngboki* sp. n.**
–	Major apical lobe of genitalia with distinctly wider apical margin (Fig. 3I)	***Pseudoexeirarthra hlavaci* sp. n.**

### Pseudoexeirarthra spinifer

Taxon classificationAnimaliaColeopteraStaphylinidae

(Broun)

**Figure 4.**
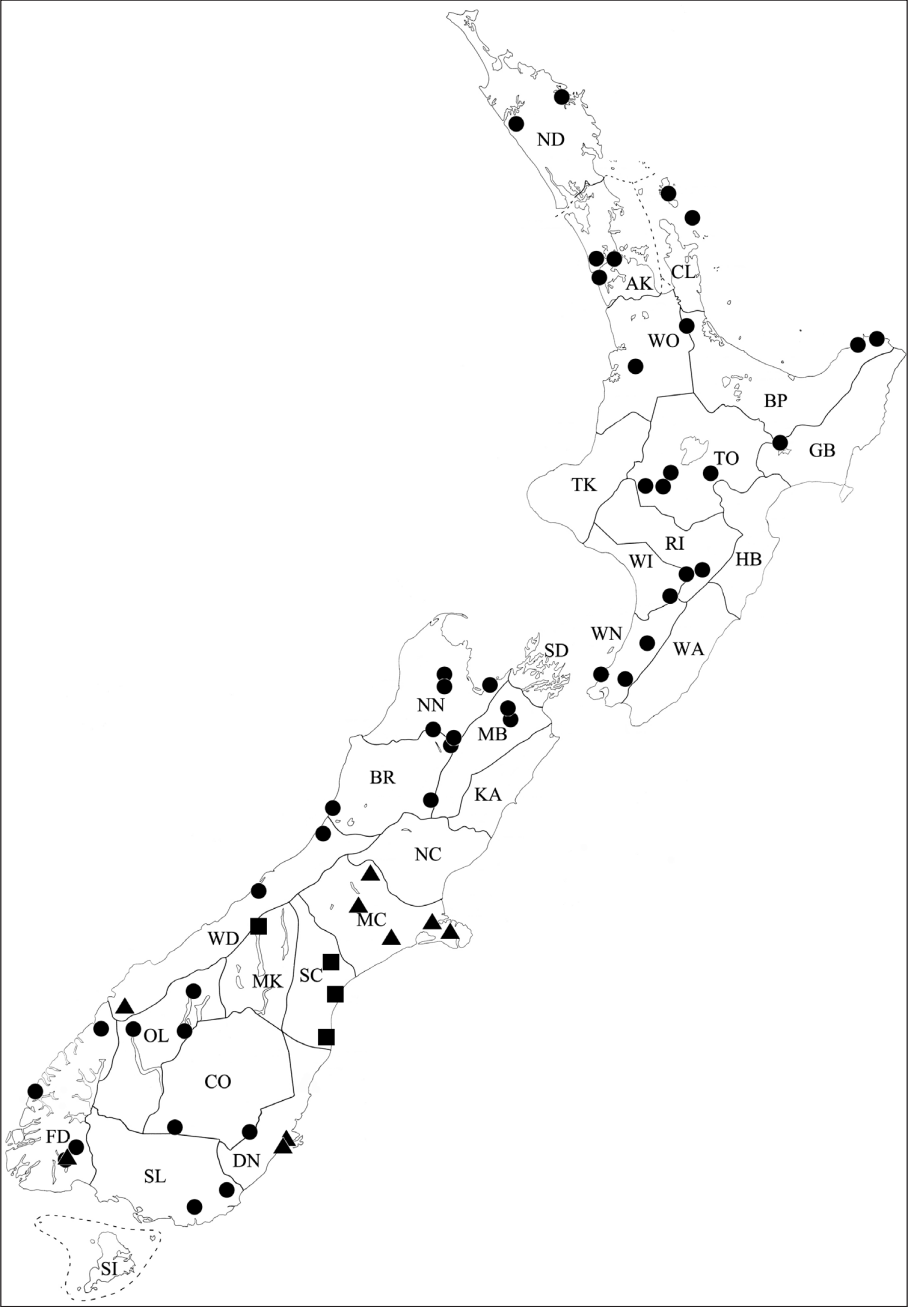
Known collection localities of *Pseudoexeirarthra* gen. n. *Pseudoexeirarthra spinifer* (Broun): black circles; *Pseudoexeirarthra colorata* (Broun): black triangles; *Pseudoexeirarthra puncticollis* (Broun): black squares.

Figs 1A , 2 , 3A , 4

Sagola spinifer Broun, 1895: 75. Hudson 1923: 365; 1934: 183. Raffray 1924: 233. Newton and Thayer 2005. Nomura and Leschen 2006: 244.
Sagola dilucida Broun, 1914: 157. Hudson 1923: 365; 1934: 184. Newton and Thayer 2005. Nomura and Leschen 2006: 242. **syn. n.**
Sagola guinnessi Broun, 1911: 502. Hudson 1923: 365; 1934: 184. Raffray 1924: 232. Newton and Thayer 2005. Nomura and Leschen 2006: 242. **syn. n.**
Sagola longicollis Broun, 1911: 498. Hudson 1923: 365; 1934: 183. Raffray 1924: 232. Newton and Thayer 2005. Nomura and Leschen 2006: 243. **syn. n.**
Sagola longula Broun, 1912: 625. Hudson 1923: 365; 1934: 183. Raffray 1924: 232. Newton and Thayer 2005. Nomura and Leschen 2006: 243. **syn. n.**
Sagola rectipennis Broun, 1921: 489. Hudson 1923: 365; 1934: 184. Newton and Thayer 2005. Nomura and Leschen 2006: 243. **syn. n.**

#### Type material.

**Lectotype. New Zealand: Waikato (WO):** 1♂ (BMNH), glued on rectangular card, “2723.” [white label, handwritten]; “Mount. Pirongia” [white label, printed]; “New Zealand Broun Coll. Brit. Mus. 1922-482.” [white label, printed]; “Sagola spinifer” [white label, handwritten]; “LECTOTYPE ***Pseudoexeirarthra spinifer*** (Broun) Desig. Park and Carlton 2013” [red label, printed]. *The lectotype designation is required because Broun did not explicitly designate a type specimen, and his comments suggest that three specimens were examined (Broun, 1875: 75). This designation will fix the identity of the species and facilitate its recognition by future workers. **Paralectotypes (1 male, 1 female). New Zealand: Waikato (WO):** 1♀ (BMNH), glued on rectangular card, “Type” [red label, printed]; “2723.” [white label, handwritten]; “Pirongia” [white label, printed]; “New Zealand Broun Coll. Brit. Mus. 1922-482.” [white label, printed]; “Sagola spinifer” [white label, handwritten]; “PARALECTOTYPE ***Pseudoexeirarthra spinifer*** (Broun) Desig. Park and Carlton 2013” [yellow label, printed]. 1♂ (NZAC), glued on rectangular card, “Pirongia” [white label, printed]; “var. 2723.” [white label, handwritten]; “Sagola spinifer antennae.” [white label, handwritten]; “Broun” [white label, handwritten]; “ N.Z. Arthropod Collection, NZAC Private Bag 92710 New Zealand” [yellow label, printed]; “PARALECTOTYPE ***Pseudoexeirarthra spinifer*** (Broun) Desig. Park and Carlton 2013” [yellow label, printed].

**Holotype of *Sagola dilucida*: New Zealand: Auckland (AK):** 1♀ (BMNH), glued on rectangular card, “Type” [red label, printed]; “New Zealand Broun Coll. Brit. Mus. 1922-482.” [white label, printed]; “Epsom. Jany.1912.” [white label, handwritten]; “3520.♂” [white label, handwritten]; “Sagola dilucida” [white label, handwritten]. The original label indicates the specimen is male, but it is female.

**Holotype of *Sagola guinnessi*: New Zealand: Taupo (TO):** 1♂ (BMNH), glued on rectangular card, “Type” [red label, printed]; “3373.” [white label, handwritten]; “New Zealand Broun Coll. Brit. Mus. 1922-482.” [white label, printed]; “Erua. 5.3.1912.” [white label, handwritten]; “Sagola guinnessi.” [white label, handwritten]. The original label indicates the specimen is female, but it is male.

**Holotype of *Sagola longicollis*: New Zealand: Taupo (TO):** 1♀ (BMNH), glued on rectangular card, “Type” [red label, printed]; “3369” [white label, handwritten]; “New Zealand Broun Coll. Brit. Mus. 1922-482.” [white label, printed]; “Mahuia. Jany.1911.” [white label, handwritten]; “Sagola longicollis.” [white label, handwritten].

**Holotype of *Sagola longula*: New Zealand: Auckland (AK):** 1♀ (BMNH), glued on rectangular card, “Type” [red label, printed]; “15.” [white label, handwritten]; “New Zealand Broun Coll. Brit. Mus. 1922-482.” [white label, printed]; “auckland. N.Z. Lawson” [white label, handwritten]; “Sharp Coll. 1905-313.” [white label, printed]; “Sagola longula.” [white label, handwritten].

**Syntype of *Sagola rectipennis*: New Zealand: Otago Lakes (OL):** 1♂ (BMNH), glued on rectangular card, “3997.♂” [white label, handwritten]; “New Zealand Broun Coll. Brit. Mus. 1922-482.” [white label, printed]; “Mt. Alfred. 9.2.1914” [white label, handwritten]; “Sagola ♂. rectipennis” [white label, handwritten]. 1♂ (BMNH), glued on rectangular card, “3997.♂” [white label, handwritten]; “New Zealand Broun Coll. Brit. Mus. 1922-482.” [white label, printed]; “Mt. Alfred. 9.2.1914” [white label, handwritten]; “Sagola rectipennis” [white label, handwritten]. 1♂ (BMNH), glued on rectangular card, “3997.♂” [white label, handwritten]; “New Zealand Broun Coll. Brit. Mus. 1922-482.” [white label, printed]; “Mt. Alfred. 9.2.1914” [white label, handwritten].

#### Additional material

**(n = 198; 95 males, 103 females). New Zealand: Auckland (AK):** 1♂ 1♀, Waitakere Ra, Cascade-Kauri Park, Up. Kauri tr, 170 m, 8 XII 1984–25 I 1985, kauri-podo-hdwd, A. Newton, M. Thayer 680, FIT&window trap (DSC); 1♂, Lynfield, Tropicana dr, 14 VIII 1976, G. Kuschel, litter (NZAC); 1♂ 1♀, Lynfield, 7 IX 1980, G. Kuschel, litter (NZAC); 2♀♀, Woodhill, 27 II 1976, C. F. Butcher, pit trap (NZAC); 1♂, Lynfield, 16 IV 1977, G. Kuschel (NZAC); **Bay of Plenty (BP):** 1♀, Orete Forest, Te Puia Hut, 230 m, 29 I 1993, R. M. Emberson, litter (NZAC); 1♂1♀, Lottin Pt Rd, Waenga Bush, 24 XI 1992–29 I 1993, R. C. Henderson, Malaise trap (NZAC); 1♂, Lottin Pt Rd, Waenga, 27 I 1993, R. C. Henderson, litter (NZAC); 1♂, Te Koau, Main Ridge, 220 m, 23 IX 1992, J. S. Dugdale, litter (NZAC); 1♂, Te Koau, Twin Puriri’s, 31 I–15 III 1993, R. C. Henderson, pit trap (NZAC); 1♂, Papatea, 13 X–23 XI 1992, G. Hall, pit trap (NZAC); 1♂, Mamaku Ra, 18 I 1972, G. W. Ramsay, litter (NZAC); 1♀, Te Koau, Bush Track, 23 IX 1992, J. S. Dugdale, litter (NZAC); 1♀, Mount Te Aroha, summit, 19 XI 2005, J. Nunn, moss (NZAC); 1♀, Kaimai-Mamaku Forest Park, Mt. Te Aroha summit rd, 450 m, 37°31.43'S, 175°44.01'E, 19 XI 2005, mixed broadleaf forest with many tree ferns, nikau palms, FMHD#2005-016, FIT, A. Newton, M. Thayer, ANMT site 1144 (FMNH); **Buller (BR):** 5♂♂ 6♀♀, Nelson Lakes NP, Mt. Robert, Speargrass tr, 875 m, 41°49.47'S, 172°48.31'E, 17 XII 2005, *Nothofagus* forest, FMHD#2005-110, litter, A. Solodovnikov, D. Clarke, ANMT site 1161 (FMNH); 22♂ 12♀♀, (1♂, slide-mounted), Lewis Pass NR, 11.9 km ese Spring Junction, 540 m, 17 XII 1984–21 I 1985, *Nothofagus* forest, A. Newton, M. Thayer, 715, window trap (FMNH); 8♂♂ 14♀♀, Nelson Lakes NP, Lake Rotoroa, Braeburn tr, 470 m, 16 XII 1984–7 I 1985, *Nothofagus* forest, A. Newton, M. Thayer 712, window trap (FMNH); 4♂♂ 8♀♀, Nelson Lakes NP, n slope Mt. Robert, Pinchgut tr, 950 m, 14 XII 1984–6 I 1985, *Nothofagus* forest, A. Newton, M. Thayer 707, window trap (FMNH&DSC); 2♀♀, Nelson Lakes NP, n slope Mt. Robert, Pinchgut tr, 950 m, 14 XII 1984–6 I 1985, *Nothofagus* forest, A. Newton, M. Thayer 707, litter (FMNH); 1♂2♀♀, Nelson Lakes NP, Lake Rotoiti, St. Arnaud tr, 645 m, 14 XII 1984–6 I 1985, *Nothofagus* forest, A. Newton, M. Thayer 705, window trap (FMNH); 1♂, Nelson Lakes NP, Mt. Robert Rd, 660 m, 26 XII 1984–6 I 1985, *Leptospermum*-*Nothofagus* forest, A. Newton, M. Thayer 722, FIT&window trap (DSC); 1♂, Lewis Pass NR, 13.2 km s Lewis Pass, 650 m, 17 XII 1984–21 I 1985, *Nothofagus* forest, A. Newton, M. Thayer 713, FIT&window trap (DSC); 2♂♂, Mt. Misery, Ecology Div Stn, 460 m, 24–26 I 1977, J. S. Dugdale, water trap (NZAC); 1♀, Greymouth, Boddytown, 8 II 1984, J.C. Watt, litter (NZAC); 2♂♂, Rd to Mt. Robert, 762 m, Lake Rotoiti, 3 V 1966, J. I. Townsend, moss (NZAC); **Central Otago (CO):** 1♂ 1♀, Waipori, 610 m, Stony Stream, 2 XI 1979, J. C. Watt, moss (NZAC); 1♀, Piano Flat, Waikaia Forest, 25 IV 2007, washed soil beech forest (JTN); **Coromandel (CL):** 2♀♀, Cuvier I, Northwest Ridge, 25 II–2 III 1982, G. Hall, malaise trap (NZAC); 1♂, Cuvier I, 25 II–2 III 1982, G. Hall, pit trap (NZAC); 1♂, Great Barrier I, Little Windy Hill, 100 m, 25 II–19 III 2003, K. Parsons, malaise trap (AMNZ); 1♂ 3♀♀, Great Barrier I, Little Windy Hill, 13 XII 2002–17 I 2003, P. Sutton, Malaise (AMNZ); 1♂ 1♀, Cuvier I, Ridge Tr, 100 m, 10–18 XI 1999, J. W. Early, S. E. Thorpe, malaise trap (AMNZ); 1♂, Great Barrier I, Little Windy Hill, 17 I–27 II 2003, K. Parsons, malaise trap (AMNZ); 1♂, Great Barrier I, Little Windy Hill, 220 m, 7 XI–11 XII 2001, P. Sutton, J. Gilbert, malaise trap (AMNZ); 3♀♀, Great Barrier I, Little Windy Hill, 220 m, 11 XII 2001–18 I 2002, P. Sutton (AMNZ); 1♀, Cuvier I, Pumphouse Stream, 120 m, 14 XI 1999, J. W. Early, S.E. Thorpe, litter (AMNZ); 1♀, Great Barrier I, Little Windy Hill, 220 m, 21 II 2001–26 III 2002, P. Sutton, malaise trap (AMNZ); 1♀, Great Barrier I, Little Windy Hill, 220 m, 18 I–21 II 2002, P. Sutton, malaise trap (AMNZ); **Fiordland (FD):** 2♂♂, Lake Hauroko, Southland, 2 II 1966, J. I. Townsend, moss (NZAC); 1♂, Secretary I, Gut Bay, 24 XI 1981, C.F. Butcher, beech litter and rotten wood (NZAC); 1♂, Fiordland NP, Monowai Lake, 4km sw Monowai, 11–14 III 2010, J. W. Early, yellow pan traps, *Nothofagus solandri* forest (DSC); 1♂ 1♀, Fiordland NP, Milford Sound rd, Smithy Creek Campground area, 400 m, 44°57.07'S 168°01.16'E, 9 XII 2005, *Nothofagus fusca* & *Nothofagus menziesii* open forest, FMHD#2005-089, litter, M. Thayer, A. Newton, ANMT site 1170 (FMNH); 1♀, Secretary I, 850 m, 30 XI 1981, C.F. Butcher, pit trap (NZAC); **Gisborne (GB):** 1♂, Urewera NP, Waikaremoana rd, s end Matanunui Ridge, 720 m, 38°44.40'S, 177°05.81'E, 22 XI–23 XII 2005, mixed broadleaf (incl. *Nothofagus fusca*)-podocarp, FMHD#2005-028, FIT, M. Thayer, A. Solodovnikov, ANMT site 1149 (FMNH); 1♀, Lake Waikaremoana, 17 I 1972, G. W. Ramsay, litter; 1♀, Urewera NP, at large, #021, 6–8 III 2000, C. Carlton, A. Weir (LSAM); 1♀, Urewera NP, Lake Waikremoana, nr Caravan Park, shoreline toetoe, FIT, 23 III 2000, C. Carlton, A. Weir, #078 (LSAM); **Northland (ND):** 1♀, Paihia Opua SF, 22 I 1981, G. Kuschel, litter and rotten wood (NZAC); 1♀, Waipoua SF, Waipoua Stm, 70 m, 16–21 III 1978, S. Peck, J. Peck, malaise trap (FMNH); **Nelson (NN):** 2♂♂ 1♀, 0.6 km e Gowanbridge, 330 m, 18 XII 1984–7 I 1985, *Nothofagus* forest, A. Newton, M. Thayer 717, FIT&window trap (DSC); 1♂, Kahurangi NP, Cobb Ridge, above Cobb Reservoir, 1050 m, 41°06.35'S, 172°41.66'E, 29 XI–18 XII 2005, *Nothofagus* forest, FMHD#2005-051, FIT, A. Newton, M. Thayer, A. Solodovnikov, ANMT site 1159 (FMNH); 1♂, Kahurangi NP, Arthur Range, above Flora Saddle, 1000 m, 41°11.35'S, 172°44.46'E, 29 XI–18 XII 2005, *Nothofagus* forest, FMHD#2005-046, litter, A. Newton, M. Thayer, ANMT site 1156 (FMNH); 1♀, Cobb Ridge, east of Cobb Reservoir, 990 m, 2 I 1985, *Nothofagus* forest, A. Newton, M. Thayer 728, litter (DSC); 1♂ 1♀, Canaan Harwoods tr, 4 II 1965, L. P. Marchant, litter (NZAC); 1♂, Dovedale, 11 X 1963, J. I. Townsend, litter (NZAC); 2♀♀, Lake Rotoiti, 19 II 1965, L. P. Marchant, moss (NZAC); 1♀, Lake Rotoiti, 27 VII 1965, A. K. Walker, moss (NZAC); **Otago Lakes (OL):** 1♂ 1♀, 44.5 km nw Wanaka, 350m, Matukituki Valley, 44’29S 168’47E, #079, *Nothofagus* forest litter, 18 I 1998, C. Carlton, R. Leschen (LSAM); 1♂ 1♀, Makarora Bush, Makarora, 7–9 XI 1997, J. Nunn (JTN); 1♂ 1♀, Paradise, 2 II 1984, J. C. Watt, wood mould (NZAC); 1♂, Upper Makarora, 17 I 1968, F. A. Alack, litter (NZAC); 1♀, 10.5 km nw Glenorchy, *Nothofagus* forest, 44°47'S, 169°27'E, FIT #143, 19–24 I 1998, C. Carlton, R. Leschen (LSAM); **Rangitikei (RI):** 1♀, Ruahine Ra, Armstrong Saddle, 1370 m, 26 XII 1983, J. C. Watt, litter (NZAC); 1♀, Ruahine Ra, Triplex, 10 II 1980, C. F. Butcher, litter (NZAC); **Marlborough Sounds (SD):** 3♂♂ 2♀♀, Wairau V, Schroders Ck, 7 IX 1966, J. I. Townsend, litter 66/295 (NZAC); 1♀, Upper Wairau Valley, 731 m, 5 IX 1966, J. I. Townsend, moss (NZAC); 3♀♀, Head Fabians Valley, 23 X 1963, J. I. Townsend, litter (NZAC); 1♂, Rainbow SF, Connors Ck, 825 m, 21 XII 1981, J. W. Early, sweeping (NZAC); **Southland (SL):** 2♀♀, Catlins SF Park, 15 II 1982, C.F. Butcher, J. S. Dugdale, litter (NZAC); 1♀, Owaka Glenomaru Reserve, 18 I 1978, S. Peck, J. Peck, litter (FMNH); **Taupo (TO):** 1♂, Kaimanawa North Forest Park, 850m, 11 III 1978, J. S. Dugdale, moss (NZAC); 1♂, Kaimanawa N Forest Park Saddle, 20 II 1978, J. S. Dugdale, litter (NZAC); 1♀ (slide-mounted), Erua, 27 I 1982, C. F. Butcher, litter and moss (NZAC); 1♀, Erua, 16 XII 1961, G. Kuschel, litter (NZAC); **Westland (WD):** 1♂, Doughboy Creek, 6 km sw Mahitai, 5 II 1984, J. C. Watt, wood mould (NZAC); 1♀, Hokitika Gorge, 29 I 1978, S. Peck, litter (FMNH); 1♀, Jacksons Bay, 23 IX 1979, A. K. Walker, moss (NZAC); **Wanganui (WI):** 1♂ 1♀, nr Glow-worm Cave, Table Hill rd nr Apiti, 8 II 1997, J. Nunn (JTN); 1♂, Ashhurst Domain, Ashhurst, 23 X 1998, J. Nunn, litter (JTN); **Wellington (WN):** 3♂♂ 1♀, Mana Island, 4–6 II 1994, J. Nunn, decayed wood (JTN); 1♂, Tararua Ra, e Basin Logan, 1300 m, 6 XII 1984, R. C. Craw, turf plants (NZAC); 1♂, Pakuratahi Forks, 8 VII 1994, J. Nunn, litter (JTN); 1♀, Tararua Ra, Dundas Hut Ridge, 990 m, 13 II 1985, C. F. Butcher, litter (NZAC); 1♀, n Titahi Bay, Rocky Bay, 28 XII 1980, J. C. Watt, litter and humus (NZAC); 1♀, Tararua Forest Park, Waitewaewae tr, 220 m, 40°51.98'S, 175°15.319'E, 26 XI–21 XII 2005, broadleaf-podo forest, FMHD#2005-034, FIT, A. Newton, M. Thayer, ANMT site 1152 (FMNH); **Waikato (WO):** 1♂, Pirongia Forest Park, Mahaukura tr, 270 m, 37°58.22'S, 175°06.52'E, 18 XI–27 XII 2005, broadleaf forest, FMHD#2005-009, FIT, A. Newton, M. Thayer, et al., ANMT site 1142 (FMNH).

#### Diagnosis.

This species is distinguished from the other species of this genus by the following combination of characters: body length 2.3–2.8 mm; eyes large, as long as temples (Fig. 1A); antennomeres 3–7 subquadrate, 8–10 weakly transverse; median lobe of male genitalia divided in apical third, broadest at base (Fig. 3A); parameres asymmetrical, left much broader basally than right (Fig. 3A).

#### Redescription.

Length 2.3–2.8 mm. Body reddish brown, antennae, legs, maxillary palpi and elytra paler (Fig. 1A). Head bluntly rectangular, longer than wide, widest across eyes (Fig. 2A). Antennomere 1 approximately 1.5 times as long as wide, 2 longer than wide, 3–7 subquadrate, 8–10 weakly transverse. Eyes each large and prominent, as long as temple (Fig. 2A). Prosternum as long as wide, widest at apical one-third (Fig. 2B). Elytra longer than wide (Fig. 1A). Hind wings fully developed. Meso- metaventrites trapezoidal, longer than wide (Fig. 2C). Tergite IV with pair of transverse patches of microtrichia reaching middle. Median lobe of genitalia divided, broadest at base (Fig. 3A). Phallobase symmetrical and rounded (Fig. 3A). Parameres asymmetrical, left paramere much broader at middle than right (Fig. 3A).

#### Type locality.

Mount Pirongia (WO), New Zealand.

#### Distribution.

Auckland (AK), Bay of Plenty (BP), Buller (BR), Central Otago (CO), Coromandel (CL), Fiordland (FD), Gisborne (GB), Northland (ND), Nelson (NN), Otago Lakes (OL), Rangitikei (RI), Marlborough Sounds (SD), Southland (SL), Taupo (TO), Westland (WD), Wanganui (WI), Wellington (WN), Waikato (WO) (Fig. 4: black circles).

#### Habitat.

Most specimens were collected using malaise, flight intercept, window traps, or by sifting leaf litter in broadleaf, podocarp, hardwood and *Nothofagus* forests.

#### Comments.

Specimens of *Pseudoexeirarthra spinifer* can be separated from those of the other species by the large eyes, fully developed hind-wings, shapes of antennomeres, and genitalia. The type specimens of *Sagola dilucida*, *Sagola guinnessi*, *Sagola longicollis*, *Sagola longula* and *Sagola rectipennis* share these diagnostic characters. For these reasons, we have placed these names in synonymy with *Pseudoexeirarthra spinifer*.

### Pseudoexeirarthra colorata

Taxon classificationAnimaliaColeopteraStaphylinidae

(Broun)

Figs 1B , 3B , 4

Sagola colorata Broun, 1914: 156. Hudson 1923: 365; 1934: 184. Newton and Thayer 2005. Nomura and Leschen 2006: 241.
Sagola insueta Broun, 1914: 157. Hudson 1923: 365; 1934: 184. Newton and Thayer 2005. Nomura and Leschen 2006: 242. **syn. n.**

#### Type material.

**Holotype. New Zealand: Mid Canterbury (MC):** 1♀ (BMNH), glued on rectangular card, “Type” [red label, printed]; “3519.” [white label, handwritten]; “New Zealand Broun Coll. Brit. Mus. 1922-482.” [white label, printed]; “McClennans. 25.3.1912.” [white label, handwritten]; “Sagola colorata.” [white label, handwritten].

**Holotype of *Sagola insueta*. New Zealand: Mid Canterbury (MC):** 1♀ (BMNH), glued on rectangular card, “Type” [red label, printed]; “3521.” [white label, handwritten]; “New Zealand Broun Coll. Brit. Mus. 1922-482.” [white label, printed]; “Rakaia. 6.7.1912.” [white label, handwritten]; “Sagola insueta” [white label, handwritten].

#### Additional material

**(n = 23; 21 males, 2 females). New Zealand: Dunedin (DN):** 1♂, Nicol Creek, Leith Valley, 25 V 2006, J. Nunn, litter (JTN); 1♂, Vauxhall, 13 IX 2001, J. Nunn, FIT (JTN); 1♂, The Tunnels, Silverpeaks, 2 VI 2001, J. Nunn, moss and litter (JTN); 1♂, Vauxhall, 26 II 2011, 45°54.24'S, 170°31.89'E, 167 m, J. Nunn, washes soil (JTN); 1♂, Town Belt, 26 VII 1997, J. Nunn, decayed wood (JTN); 1♂, Vauxhall, 27 I 2000, J. Nunn, FIT (JTN); **Fiordland (FD):** 6♂ 1♀, Lake Hauroko, 2 XI 1966, J. I. Townsend, litter (NZAC); 1♂, Hollyford Camp, 10 XII 1966, A. K. Walker, litter (NZAC); **Mid Canterbury (MC):** 7♂♂, Banks Peninsula, Hay Scenic Res, Pigeon Bay, 25 m, 11 XII 1984–22 I 1985, Podocarp-hdwd forest, A. Newton, M. Thayer 702, window trap (FMNH); 1♂, Craigieburn SF, 850 m, #023, 42°10.8'S, 171°42'E, *Nothofagus* litter, 9 I 1998, C. Carlton, R. Leschen (LSAM); 1♀, McLellans Bush, Mt. Hutt, 11 I 2008, J. Nunn, on sooty mould on beech bole (JTN).

#### Diagnosis.

This species is distinguished from the other species of this genus by the following combination of characters: body length 2.2–2.5 mm; eyes large, two-thirds length of temples (Fig. 1B); antennomeres 2–7 longer than wide, 8–10 subquadrate; median lobe of male genitalia divided, minor lobe longer than major lobe and covered with small tubercles (Fig. 3B); parameres symmetrical, setae present from apices to middle (Fig. 3B).

#### Redescription.

Length 2.2–2.5 mm. Body reddish brown, antennae, legs, maxillary palpi and elytra paler (Fig. 1B). Head rectangular, longer than wide, widest across eyes (Fig. 1B). Antennomere 1 approximately 1.5 times longer than wide, 2–7 longer than wide, 8–10 subquadrate. Eyes large and prominent, two-thirds length of temples. Prosternum as long as wide, widest at apical one-third. Elytra longer than wide (Fig. 1B). Hind wings fully developed. Meso- and metaventrites trapezoidal, longer than wide. Tergite IV with pair of transverse patches of microtrichia reaching middle. Median lobe of genitalia divided, minor lobe longer than major lobe and covered with small tubercles (Fig. 3B). Phallobase symmetrical and rounded (Fig. 3B). Parameres symmetrical, setae present from apices to middle (Fig. 3B).

#### Type locality.

McClennan’s Bush, near Methven (MC), New Zealand.

#### Distribution.

Dunedin (DN), Fiordland (FD), Mid Canterbury (MC) (Fig. 4: black triangles).

#### Habitat.

Specimens were collected using flight intercept traps, and by soil washing or sifting leaf and wood litter.

#### Comments.

The type specimen of *Sagola insueta* shares the diagnostic characters of *Pseudoexeirarthra colorata*. For this reason, we have placed *Sagola insueta* in synonymy with *Pseudoexeirarthra colorata*.

### Pseudoexeirarthra puncticollis

Taxon classificationAnimaliaColeopteraStaphylinidae

(Broun)

Figs 1C , 3C , 4

Sagola puncticollis Broun, 1911: 499. Hudson 1923: 365; 1934: 183. Raffray 1924: 232. Newton and Thayer 2005. Nomura and Leschen 2006: 243.

#### Type material.

**Holotype. New Zealand: South Canterbury (SC):** 1♂ (BMNH), glued on rectangular card, “Type” [red label, printed]; “3370.” [white label, handwritten]; “New Zealand Broun Coll. Brit. Mus. 1922-482.” [white label, printed]; “Timaru. -Wallace.” [white label, hand written]; “Sagola puncticollis” [white label, handwritten].

#### Additional material

**(n = 34; 29 males, 5 females). New Zealand: Mackenzie (MK):** 8♂♀, White Horse Hill, Mt. Cook, 26 X 2009, J. Nunn, litter in podocarp forest (JTN); 1♂, White Horse Hill, Mt. Cook, 25 X 2009, J. Nunn, litter in podocarp grove (JTN); **South Canterbury (SC):** 18♂♀, Gunns Bush, Waimate, 23 XII 2006, washed soil in broadleaf forest, J. Nunn (JTN); 1♂ 1♀, Orari Gorge SR, Geraldine, 7 VI 2009, J. Nunn, washed soil in broadleaf forest (JTN); 1♀ (slide-mounted), Pioneer Park, Raincliff, 9 VIII 2009, J. Nunn, washed soil in totara and kahikatea forest (JTN); 1♂, Kelceys Bush, Waimate, 20 I 1966, J. I. Townsend, moss; 1♀, Mt. Dalgety, 1737 m, 19 I 1966, G. W. Ramsay, J. I. Townsend, moss (NZAC).

#### Diagnosis.

This species is distinguished from the other species of this genus by the following combination of characters: body length 2.3–2.6 mm; eyes one-half lengths of temples (Fig. 1C); antennomeres 2–5 longer than wide, 6–10 subquadrate; median lobe of male genitalia divided, major lobe triangular, minor lobe slightly longer and unequally laterally tuberculate near apex (Fig. 3C); parameres nearly symmetrical, setae present from apices to middle (Fig. 3C).

#### Redescription.

Length 2.3–2.6 mm. Body reddish brown, antennae, legs, maxillary palpi and elytra paler (Fig. 1C). Head round, as long as wide, widest across eyes (Fig. 1C). Antennomere 1 approximately 1.5 times longer than wide, 2–5 longer than wide, 6–10 subquadrate. Eyes one-half lengths of temples. Prosternum as long as wide, widest at apical one-third. Elytra longer than wide (Fig. 1C). Hind wings fully developed. Meso- and metaventrites trapezoidal, longer than wide. Tergite IV with pair of transverse patches of microtrichia reaching middle. Median lobe of genitalia divided, apical lobe triangular, minor lobe slightly longer covered with tubercles (Fig. 3C). Phallobase symmetrical and rounded (Fig. 3C). Parameres symmetrical, setae present from apices to middle (Fig. 3C).

#### Type locality.

Timaru (SC), New Zealand.

#### Distribution.

Mackenzie (MK), South Canterbury (SC) (Fig. 4: black squares).

#### Habitat.

Specimens were collected by soil washing and sifting moss litter in broadleaf or podocarp forests.

### Pseudoexeirarthra sungmini sp. n.

Taxon classificationAnimaliaColeopteraStaphylinidae

http://zoobank.org/3FF61CCF-3FB8-440D-9579-E83F7C5EED9B

Figs 1D , 3D , 5

#### Type material.

**Holotype. New Zealand: Nelson (NN):** 1♂ (NZAC), aedeagus dissected and mounted in balsam on a clear plastic card, “NEW ZEALAND NN Mt Arthur/Flora Sdl Tck, c1400m 28-Nov-05”, “On mossy overhangs by gully. D Clarke, J Nunn”, “HOLOTYPE ***Pseudoexeirarthra sungmini*** Park and Carlton des. 2013”. **Paratype (1 male): New Zealand: Nelson (NN):** 1♂, Cobb, L. Sylvester, 1329 m, 31 III 1969, J. S. Dugdale, litter (NZAC).

#### Etymology.

This species is named after Dr. Sung Min Boo, Professor of Biology, Chungnam National University (Daejeon, South Korea), world algal systematist and, an enthusiastic supporter of this study.

#### Diagnosis.

**Figure 5.**
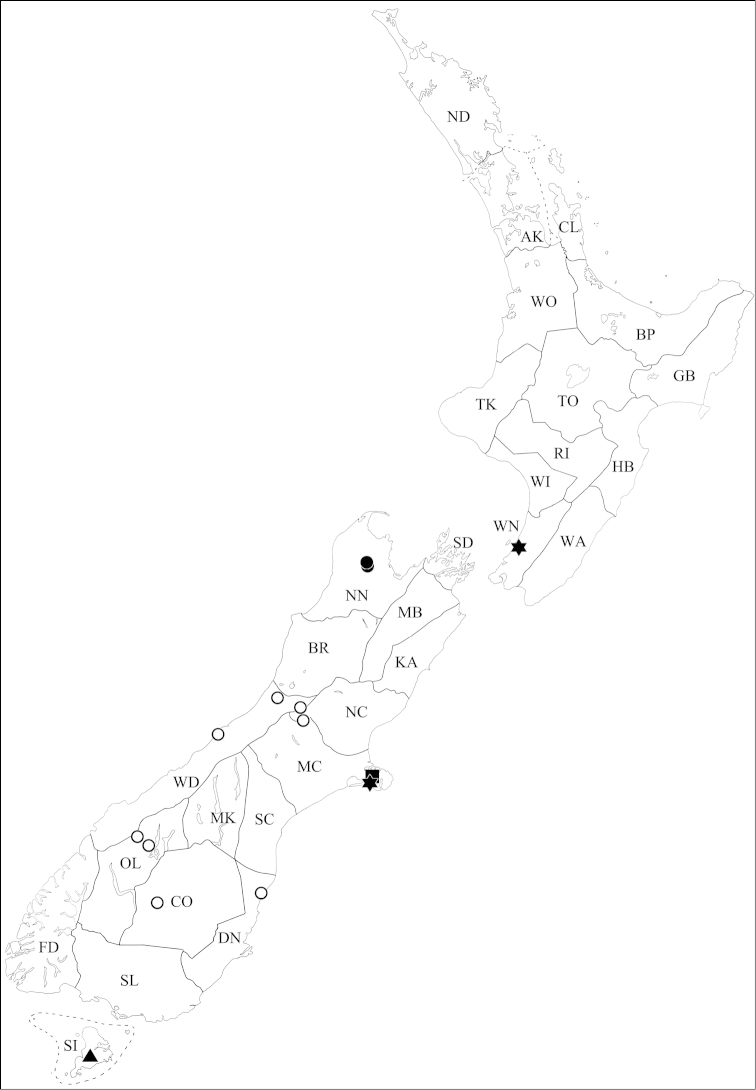
Known collection localities of *Pseudoexeirarthra* gen. n. *Pseudoexeirarthra sungmini* sp. n.: black circles; *Pseudoexeirarthra kwangguki* sp. n.: black triangle; *Pseudoexeirarthra youngboki* sp. n.: black square; *Pseudoexeirarthra seiwoongi* sp. n.: black stars; *Pseudoexeirarthra parkeri* sp. n.: white circles.

This species is distinguished from other the species of this genus by the following combination of characters: body length 1.9–2.1 mm; eyes two-thirds length of temples (Fig. 1D); antennomeres 2–4 longer than wide, 5–7 subquadrate, 8–10 weakly transverse; median lobe of male genitalia divided, broadest near base, major lobe short with semicircular depression anteriorly, minor lobe triangular and longer (Fig. 3D); parameres symmetrical, setae limited to apical fourth (Fig. 3D); only known from Nelson (Fig. 5: black circles).

#### Description of male.

Length 1.9–2.1 mm. Body reddish brown, antennae, legs, maxillary palpi and elytra paler (Fig. 1D). Head round, as long as wide, widest across eyes (Fig. 1D). Antennomere 1 approximately 1.5 times longer than wide, 2–4 longer than wide, 5–7 subquadrate, 8–10 weakly transverse. Eyes two-thirds length of temples. Prosternum as long as wide, widest at one-third length. Elytra longer than wide (Fig. 1D). Hind wings fully developed. Meso- and metaventrites trapezoidal, longer than wide. Tergite IV with pair of transverse patches of microtrichia reaching middle. Median lobe of genitalia divided, broadest near base, major lobe short with semicircular depression anteriorly, minor lobe triangular and longer (Fig. 3D). Phallobase symmetrical and rounded (Fig. 3D). Parameres nearly symmetrical, setae limited to apical fourth (Fig. 3D).

#### Distribution.

Nelson (NN) (Fig. 5: black circles).

#### Habitat.

Specimens were collected by sifting moss and leaf litter.

### Pseudoexeirarthra kwangguki sp. n.

Taxon classificationAnimaliaColeopteraStaphylinidae

http://zoobank.org/5F206EEA-7E3C-4153-931E-905C0A1D1164

Figs 1E , 3E , 5

#### Type material.

**Holotype. New Zealand: Stewart Island (SI):** 1♂ (NZAC), aedeagus dissected and mounted in balsam on a clear plastic card, “**New Zealand: SI:** Table Hill, 366–610m 15 II 1968, G. Kuschel Moss 68/52”, “HOLOTYPE ***Pseudoexeirarthra kwangguki*** Park and Carlton des. 2013”. **Paratypes (2 females): New Zealand: Stewart Island (SI):** 2♀♀ (1♀, slide-mounted), same data as holotype (NZAC).

#### Etymology.

This species is named for Dr. Kwang-Guk An, Professor of Biology, Chungnam National University (Daejeon, South Korea), freshwater ecosystem specialist, and an enthusiastic supporter of this study.

#### Diagnosis.

This species is distinguished from the other species of this genus by the following combination of characters: body length 1.8–2.0 mm; eyes one-half lengths of temples (Fig. 1E); elytra subquadrate (Fig. 1E); hind wings represented by small pads; tergite IV without patch of microtrichia (Fig. 1E); antennomeres 3–6 subquadrate, 7–10 weakly transverse; median lobe of male genitalia deeply divided (Fig. 3E); parameres nearly symmetrical, setae limited to apical fourth (Fig. 3E); known from Stewart Island (Fig. 5: black triangle).

#### Description.

Length 1.8–2.0 mm. Body reddish brown, antennae, legs, maxillary palpi and elytra paler (Fig. 1E). Head round, as long as wide, widest across eyes (Fig. 1E). Antennomere 1 approximately 1.5 times longer than wide, 2 longer than wide, 3–6 subquadrate, 7–10 weakly transverse. Eyes one-half length of temples. Prosternum as long as wide, widest at one-third length. Elytra as long as wide (Fig. 1E). Hind wings represented by small pads. Meso- metaventrites trapezoidal, as long as wide. Tergite IV without patch of microtrichia. Median lobe deeply divided (Fig. 3E). Phallobase symmetrical and rounded (Fig. 3E). Parameres symmetrical, setae at apical fourth (Fig. 3E).

#### Distribution.

Stewart Island (SI) (Fig. 5: black triangle).

#### Habitat.

Specimens were collected by sifting moss litter.

### Pseudoexeirarthra youngboki sp. n.

Taxon classificationAnimaliaColeopteraStaphylinidae

http://zoobank.org/18C67D5D-A05D-4874-9AF2-F69ACB9F562C

Figs 1F , 3F , 5

#### Type material.

**Holotype. New Zealand: Mid Canterbury (MC):** 1♂ (NZAC), aedeagus dissected and mounted in balsam on a clear plastic card, “**NEW ZEALAND: MC:** Banks Peninsula, Ahuriri Scen. Res., 450m, 43°39.971'S, 172°37.427'E, 3 XII 2005, mixed broadleaf w/emergent podocarp; FMHD#2005-069, berl., leaf & log litter, A. Newton, M. Thayer & A. Solodovnikov; ANMT site 1162”, “HOLOTYPE ***Pseudoexeirarthra youngboki*** Park and Carlton des. 2013”. **Paratypes (n = 18; 8 males, 10 females): New Zealand: Mid Canterbury (MC):** 6♂♂ 6♀♀, same data as holotype (FMHD); 1♂, Banks Peninsula, Ahuriri SR, 450 m, 43°39.971'S, 172°37.427'E, 3–6 XII 2005, mixed broadleaf w/emergent podocarp, FMHD#2005-069, FIT, A. Newton, M. Thayer, ANMT site 1162 (FMHD); 4♀♀, Bank Peninsula, Mt. Sinclair SR, 775 m, 43°42.977'S, 172°51.098'E, 3–16 XII 2005, ridgetop mixed broadleaf w/emergent *Podocarpus tatara*, FMHD#2005-070, FIT, A. Newton, M. Thayer, ANMT site 1163 (FMNH); 1♂, Prices Valley, 3–24 IV 1981, J. W. Early, yellow pan trap (LUNZ).

#### Etymology.

This species is named after Dr. Young Bok Cho, curator of Natural History Museum of Hannam University (Daejeon, South Korea), carrion and rove beetles specialist, and an enthusiastic supporter of this study.

#### Diagnosis.

This species is distinguished from the other species of this genus by the following combination of characters: body length 1.9–2.2 mm; eyes large, two-thirds lengths of temples (Fig. 1F); antennomeres 2–8 longer than wide, 9–10 weakly transverse; median lobe of male genitalia divided, apex of major lobe rectangular, minor lobe thin, longer than major lobe and bearing small tubercles (Fig. 3F); parameres symmetrical, but right slightly longer than left with setae from before midpoint apices (Fig. 3F).

#### Description.

Length 1.9–2.2 mm. Body reddish brown, antennae, legs, maxillary palpi and elytra paler (Fig. 1F). Head round, as long as wide, widest across eyes (Fig. 1F). Antennomere 1 approximately 1.5 times longer than wide, 2–8 longer than wide, 9–10 weakly transverse. Eyes large and prominent, two-thirds length of temples. Prosternum as long as wide, widest at one-third length. Elytra longer than wide (Fig. 1F). Hind wings fully developed. Meso- metaventrites trapezoidal, longer than wide. Tergite IV with pair of transverse patches of microtrichia reaching middle. Median lobe of genitalia divided, apex of major lobe rectangular, minor thin, lobe longer than major lobe and bearing small tubercles (Fig. 3F). Phallobase symmetrical and rounded (Fig. 3F). Parameres nearly symmetrical, with right slightly longer than left, with setae extending from apices to anterior to midpoints (Fig. 3F).

#### Distribution.

Mid Canterbury (MC) (Fig. 5: black square).

#### Habitat.

Specimens were collected using flight intercept or yellow pan traps, or by sifting moss and leaf litter in broadleaf and podocarp forests.

### Pseudoexeirarthra seiwoongi sp. n.

Taxon classificationAnimaliaColeopteraStaphylinidae

http://zoobank.org/18C2A952-BD29-431A-A32E-71E550D0DB48

Figs 1G , 3G , 5

#### Type material.

**Holotype. New Zealand: Wellington (WN):** 1♂ (NZAC), aedeagus dissected and mounted in balsam on a clear plastic card, “NEW ZEALAND WN 4 km along Waiotauru Rd. 16/11/91 Tararua FP”, “1159”, “HOLOTYPE ***Pseudoexeirarthra seiwoongi*** Park and Carlton des. 2013”. The original label does not record who collected the specimen, but it was collected by J. Nunn. **Paratype (1 male): New Zealand: Mid Canterbury (MC):** 1♂, Banks Penin., Peraki Saddle Scen Res, 500 m, 11 XII 1984, hdwd-podo.elfin forest, A. Newton, M. Thayer 701, log and leaf litter (FMNH).

#### Etymology.

This species is named after Dr. Sei-Woong Choi, Professor at Mokpo National University (Mokpo, South Korea), world moth specialist, and an enthusiastic supporter of this study.

#### Diagnosis.

This species is distinguished from the other species of this genus by the following combination of characters: body length 1.9–2.1 mm; eyes large, as long as temples (Fig. 1G); antennomeres 2–7 longer than wide, 8 subquadrate, 9–10 weakly transverse; median lobe of male genitalia divided, major lobe with triangular apex, minor lobe longer with small tubercles (Fig. 3G); parameres nearly symmetrical, but right paramere slightly longer than left with setae from apices to midpoints (Fig. 3G).

#### Description of male.

Length 1.9–2.1 mm. Body reddish brown, antennae, legs, maxillary palpi and elytra paler (Fig. 1G). Head bluntly rectangular, longer than wide, widest across eyes (Fig. 1G). Antennomere 1 approximately 1.5 times longer than wide, 2–7 longer than wide, 8 subquadrate, 9–10 weakly transverse. Eyes large and prominent, as long as temples. Prosternum as long as wide, widest at one-third length. Male elytra longer than wide (Fig. 1G). Hind wings fully developed. Meso- metathorax trapezoidal, longer than wide. Male tergite IV with pair of transverse patches of microtrichia reaching middle. Median lobe of genitalia divided, major lobe triangular apically, minor lobe longer with small tubercles (Fig. 3G). Phallobase symmetrical and rounded (Fig. 3G). Parameres nearly symmetrical, but right slightly longer than left with setae from apices to midpoints (Fig. 3G).

#### Distribution.

Mid Canterbury (MC), Wellington (WN) (Fig. 5: black stars).

#### Habitat.

The paratype was collected by sifting leaf and moss litter.

### Pseudoexeirarthra parkeri sp. n.

Taxon classificationAnimaliaColeopteraStaphylinidae

http://zoobank.org/A3BAFA02-D9AE-4179-A0D7-551EA676B919

Fig. 1H , 3H , 5

#### Type material.

**Holotype. New Zealand: Dunedin (DN):** 1♂ (NZAC), aedeagus dissected and mounted in balsam on a clear plastic card, “NEW ZEALAND DN Rocklands 21 Nov 1981 C.F. Butcher”, “Sweeping tussock nr stream”, “N.Z. Arthropod Collection, NZAC Private Bag 92170 AUCKLAND New Zealand”, “HOLOTYPE ***Pseudoexeirarthra parkeri*** Park and Carlton des. 2013”. **Paratypes (n = 16; 12 males, 4 females): New Zealand: Central Otago (CO):** 2♂♂, Carrick Range, Watts Rock, 1400 m, 11 III 1979, J. C. Watt, litter (NZAC); **Mid Canterbury (MC):** 1♂ 2♀♀, Bealy Spur, 750 m, 1 VI 1981, C. A. Muir, moss and rotten logs (NZAC); **Otago Lakes (OL):** 4♂♂, E Matukituki V, 400 m, J. W. Early, 30 I–4 II 1987, yellow pan tap (LUNZ); 1♂, Mt. Aspiring NP, Glacier Burn, 30 I 1987, J. W. Early, 400 m, sweeping *Nothofagus* forest (LUNZ); **Westland (WD):** 3♂♂ 1♀ (1♀, slide-mounted), Klondyke Corner, Arthurs Pass, 25 X 1970, D. S. Horning, litter (NZAC); 1♂, Mt. Tuhua, 1067 m, e side of L. Kaniere, 20 X 1984, C. F. Butcher, litter and mats (NZAC); 1♀, Okarito Trig, 150 m, 15 I 1982, J. W. Early, sweeping ferns and kiekie in rimu forest (LUNZ).

#### Etymology.

This species is named after Dr. Joseph Parker, world pselaphine beetle specialist, and an enthusiastic supporter of this study.

#### Diagnosis.

This species is distinguished from the other species of this genus by the following combination of characters: body length 1.8–2.0 mm; eyes large, as long as temples (Fig. 1H); antennomeres 3 subquadrate, 4–5 longer than wide, 6–8 subquadrate, 9–10 weakly transverse; median lobe of male genitalia divided, apically bifurcate minor lobe longer than curved major lobe and bearing small tubercles (Fig. 3H); parameres asymmetrical, left longer than right with setae extending from apices to near bases (Fig. 3H).

#### Description.

Length 1.8–2.0 mm. Body reddish brown, antennae, legs, maxillary palpi, elytra paler (Fig. 1H). Head round, as long as wide, widest across eyes (Fig. 1H). Antennomere 1 approximately 1.5 times longer than wide, 2 longer than wide, 3 subquadrate, 4–5 longer than wide, 6–8 subquadrate, 9–10 weakly transverse. Eyes large and prominent, as long as temples. Prosternum as long as wide, widest at one-third length. Elytra longer than wide (Fig. 1H). Hind wings fully developed. Meso- metathorax trapezoidal, longer than wide. Tergite IV with pair of transverse patches of microtrichia reaching middle. Median lobe of genitalia divided, apically bifurcate minor lobe longer than curved major lobe and bearing small tubercles (Fig. 3H). Phallobase symmetrical and rounded (Fig. 3H). Parameres asymmetrical, left paramere longer than right with setae extending from apices to bases (Fig. 3H).

#### Distribution.

Central Otago (CO), Dunedin (DN), Mid Canterbury (MC), Otago Lakes (OL), Westland (WD) (Fig. 5: white circles).

#### Habitat.

Specimens were collected using yellow pan traps, by sweeping, or by sifting forest litter.

### Pseudoexeirarthra hlavaci sp. n.

Taxon classificationAnimaliaColeopteraStaphylinidae

http://zoobank.org/6DD193A1-531D-4CC8-9881-E568FF7B466A

Fig. 1I , 3I , 6

#### Type material.

**Holotype. New Zealand: Dunedin (DN):** 1♂ (NZAC), aedeagus dissected and mounted in balsam on a clear plastic card, “New Zealand DN Leith Saddle / Leith trig tck, 420m 26 Sep 2011”, “Washed soil sample. temperate cloud forest”, “NZMS 260 144: 173868 430m”, “Voucher specimen “Beetles of Dunedin” project. JT Nunn coll.”, “HOLOTYPE ***Pseudoexeirarthra hlavaci*** Park and Carlton des. 2013”. **Paratypes (n = 29; 11 males, 18 females): New Zealand: Dunedin (DN):** 2♂♂, Swampy Summit, Dunedin, 29 X 2000, J. Nunn, tussock litter (JTN); 1♂, Leith Saddle, Swampy Spur Tck, 14 X 2001, J. Nunn, surface soil (JTN); 1♂, Waitati, 2 VIII 2008, washed soil sample in broadleaf forest (JTN); 1♂, Grahams Bush, Mt Cargill, 6 XII 2002, J. Nunn, FIT (JTN); 1♀, Swampy Summit, Dunedin, 7 I 2000, J. Nunn, shrubbery (JTN); 1♀, Cloud Forest of Leith tr, 30 XI 2003, J. Nunn, moss from tree trunk (JTN); 1♀, Grahams Bush, Mt. Cargill, 9 XII 2001, J. Nunn, podocarp-kamahi-griselinia litter (JTN); 1♀, Careys Creek near Waitati, 12 IV 2008, J. Nunn, washed soil in kanuka forest (JTN); 1♀, Swallow tr, Herbert Forest, 28 VII 2007, washed soil, J. Nunn (JTN); 1♀, Waipori Falls, 17 V 1998, *Nothofagus* forest litter (JTN); **Southland (SL):** 3♂♂, Papatowai, 9 II 1989, J. Nunn (JTN); 1♂ 3♀♀, Bog Burn, Waterloo Burn tck, 4 VI 2007, J. Nunn, washed soil in *Nothofagus* forest (JTN); 1♂, Purakaunui Falls, Catlins, 24 VI 2006, J. Nunn, soil sample in *Nothofagus* forest (JTN); 1♂, Pourakina River Walk, 27 X 2002, J. Nunn, bracket fungus (JTN); 2♀♀, Princhester Base Hut, Takitimu Forest, 4 VI 2007, J. Nunn, washed soil beech forest, 425 m (JTN); 3♀♀ (1♀, slide-mounted), Blacks Gully, Blue Mtns, 390 m, 2 XII 2006, J. Nunn, washed soil in *Nothofagus* forest (JTN); 2♀♀, Tautuku, 8 III 1989, J. Nunn (JTN); 1♀, Rakahouka, 2 IX 2007, J. Nunn, washed soil (JTN).

#### Etymology.

This species is named after Mr. Peter Hlaváč, world pselaphine beetle specialist, and an enthusiastic supporter of this study.

#### Diagnosis.

This species is distinguished from the other species of this genus by the following combination of characters: body length 2.3–2.6 mm; eyes large, as long as temples (Fig. 1I); antennomere 2 longer than wide, 3 subquadrate, 4 longer than wide, 5–8 subquadrate, 9–10 weakly transverse; median lobe of male genitalia divided, major lobe truncate and broadest at apex, minor lobe longer with small tubercles (Fig. 3I); parameres nearly symmetrical, right paramere slightly longer than left with setae present on mesal margins in apical two-thirds (Fig. 3I).

#### Description.

Length 2.3–2.6 mm. Body reddish brown, antennae, legs, maxillary palpi and elytra paler (Fig. 1I). Head round, as long as wide, widest across eyes (Fig. 1I). Antennomere 1 approximately 1.5 times longer than wide, 2 longer than wide, 3 subquadrate, 4 longer than wide, 5–8 subquadrate, 9–10 weakly transverse. Eyes large and prominent, as long as temples. Prosternum as long as wide, widest at one-third length. Elytra longer than wide (Fig. 1I). Hind wings fully developed. Meso- metaventrites trapezoidal, longer than wide. Tergite IV with pair of transverse patches of microtrichia reaching middle. Median lobe of genitalia divided, major lobe truncate and broadest at apex, minor lobe longer with small tubercles (Fig. 3I). Phallobase symmetrical and rounded (Fig. 3I). Parameres nearly symmetrical, right paramere slightly longer than left with setae present on mesal margins in apical two-thirds (Fig. 3I).

#### Distribution.

**Figure 6.**
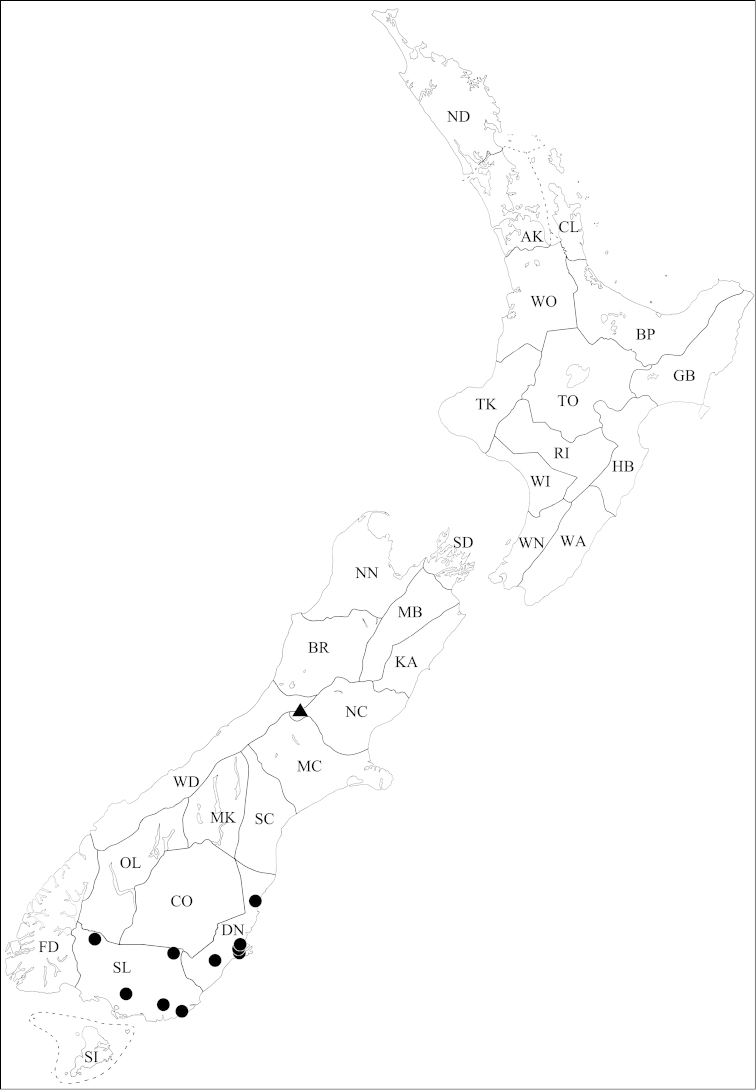
Known collection localities of *Pseudoexeirarthra* gen. n. *Pseudoexeirarthra hlavaci* sp. n.: black circles; *Pseudoexeirarthra nomurai* sp. n.: black triangle.

Dunedin (DN), Southland (SL) (Fig. 6: black circles).

#### Habitat.

Specimens were collected mostly by soil washing in *Nothofagus* and broadleaf forests.

### Pseudoexeirarthra nomurai sp. n.

Taxon classificationAnimaliaColeopteraStaphylinidae

http://zoobank.org/47CD99FE-DC17-4B90-BF07-0A3D41065E4E

Figs 1J , 3J , 6

#### Type material.

**Holotype. New Zealand: North Canterbury (NC):** 1♂ (NZAC), aedeagus dissected and mounted in balsam on a clear plastic card, “NEW ZEALAND NC Arthur Pass Kellys Creek, 8 Nov 2005 R Leschen S Nomura”, “ex litter RL1007 42°47'S 171°34'E”, “N.Z. Arthropod Collection, NZAC Private Bag 92170 AUCKLAND New Zealand”, “HOLOTYPE ***Pseudoexeirarthra nomurai*** Park and Carlton des. 2013”.

#### Etymology.

This species is named after Dr. Shûhei Nomura, co-collector of the holotype, world pselaphine beetle specialist, and an enthusiastic supporter of this study.

#### Diagnosis.

This species is distinguished from other species of this genus by the following combination of characters: body length 2.0 mm; eyes one-half length of temples (Fig. 1J); antennomeres 2–7 longer than wide, 8 subquadrate, 9–10 weakly transverse; median lobe of male genitalia divided with acute lateral spine at two-thirds length, major lobe subtruncate, minor lobe deeply bifurcate and with small tubercles (Fig. 3J); parameres nearly symmetrical, setae expending from apices to basal third (Fig. 3J).

#### Description of male.

Length 2.0 mm. Body reddish brown, antennae, legs, maxillary palpi and elytra paler (Fig. 1J). Head round, as long as wide, widest across eyes (Fig. 1J). Antennomere 1 approximately 1.5 times longer than wide, 2–7 longer than wide, 8 subquadrate, 9–10 weakly transverse. Eyes one-half length of temples. Prosternum as long as wide, widest at one-third length. Elytra longer than wide (Fig. 1J). Hind wings fully developed. Meso- metaventrites trapezoidal, longer than wide. Tergite IV with pair of transverse patches of microtrichia reaching middle. Median lobe of male genitalia divided with acute lateral spine at two-thirds length, major lobe subtruncate, minor lobe deeply bifurcate and with small tubercles (Fig. 3J). Phallobase symmetrical and rounded (Fig. 3J). Parameres nearly symmetrical, setae expending from apices to basal third (Fig. 3J).

#### Distribution.

North Canterbury (NC) (Fig. 6: black triangle).

#### Habitat.

The holotype was collected by sifting leaf litter.

## Supplementary Material

XML Treatment for Pseudoexeirarthra

XML Treatment for Pseudoexeirarthra spinifer

XML Treatment for Pseudoexeirarthra colorata

XML Treatment for Pseudoexeirarthra puncticollis

XML Treatment for Pseudoexeirarthra sungmini

XML Treatment for Pseudoexeirarthra kwangguki

XML Treatment for Pseudoexeirarthra youngboki

XML Treatment for Pseudoexeirarthra seiwoongi

XML Treatment for Pseudoexeirarthra parkeri

XML Treatment for Pseudoexeirarthra hlavaci

XML Treatment for Pseudoexeirarthra nomurai
